# Structural Mechanical Properties of 3D Printing Biomimetic Bone Replacement Materials

**DOI:** 10.3390/biomimetics8020166

**Published:** 2023-04-19

**Authors:** Xueman Lv, Shuo Wang, Zihe Xu, Xuanting Liu, Guoqin Liu, Feipeng Cao, Yunhai Ma

**Affiliations:** 1 The College of Biological and Agricultural Engineering, Jilin University, 5988 Renmin Street, Changchun 130025, China; 2 Department of Ophthalmology, China-Japan Union Hospital of Jilin University, Changchun 130031, China

**Keywords:** 3D printing, mechanical property analysis, bone scaffold, finite element method, TCP, PCL

## Abstract

One of the primary challenges in developing bone substitutes is to create scaffolds with mechanical properties that closely mimic those of regenerated tissue. Scaffolds that mimic the structure of natural cancellous bone are believed to have better environmental adaptability. In this study, we used the porosity and thickness of pig cancellous bone as biomimetic design parameters, and porosity and structural shape as differential indicators, to design a biomimetic bone beam scaffold. The mechanical properties of the designed bone beam model were tested using the finite element method (FEM). PCL/β-TCP porous scaffolds were prepared using the FDM method, and their mechanical properties were tested. The FEM simulation results were compared and validated, and the effects of porosity and pore shape on the mechanical properties were analyzed. The results of this study indicate that the PCL/β-TCP scaffold, prepared using FDM 3D printing technology for cancellous bone tissue engineering, has excellent integrity and stability. Predicting the structural stability using FEM is effective. The triangle pore structure has the most stability in both simulations and tests, followed by the rectangle and honeycomb shapes, and the diamond structure has the worst stability. Therefore, adjusting the porosity and pore shape can change the mechanical properties of the composite scaffold to meet the mechanical requirements of customized tissue engineering.

## 1. Introduction

Cancellous bone is composed of a large number of bone trabeculae interlacing with each other in a spongiform manner. It follows the principle of maximizing bone strength with minimum bone mass, and plays a role in supporting, reducing weight, cushioning, accommodating bone marrow, coping with deformation, adapting to deformation and other functions [1]. Bone cavity, joint fusion, fracture nonunion and bone defect formed after curetting bone tumor or inflammation are often filled with autogenous cancellous bone grafts in clinical practice to help bone healing. Its advantages are large osteogenic stimulation effect, fast bone healing speed and strong anti-infectivity. However, due to the limited supply of grafts, the increased incidence of infection at the donor site and some surgical complications [2,3,4], the grafts cannot meet clinical needs.

Therefore, it has always been the goal of people to seek ideal artificial bone scaffolds to replace autologous bone grafts to help bone healing [5]. The ideal artificial bone scaffold should not only be biocompatible and biodegradable, but also have certain mechanical strength, toughness, porosity and pore size close to normal human bone, which can create a microenvironment conducive to cell adhesion, growth, proliferation and function play [6,7,8]. Scaffolds that mimic natural cancellous bone are considered to have better environmental adaptability [9,10].

Among the many alternative materials, the composite material composed of polymer and bioceramic is similar to the combination of organic and inorganic components in bone tissue, which is closer to the real bone matrix environment and is widely used in bone tissue engineering research. The addition of polymers to calcium phosphate scaffolds can increase the toughness and compressive strength similar to that of bone. Similarly, the mechanical integrity and biological activity of polymers can be improved by the addition of calcium phosphate scaffolds [9,10,11,12]. There are several conventional methods for preparing porous bone scaffolds, such as solvent casting, particle leaching, freeze-drying, heat-induced phase separation and gas foaming [13]. The main defects of these methods are the existence of cytotoxic solvents, uneven pore distribution and difficult to control pore size [14].

With the appearance of additive manufacturing technology, namely, 3D printing technology, the development of bone scaffolds has taken a new direction [15,16]. The 3D printing technology can control the shape, size, internal porosity and porosity of the target structure. These techniques, combined with computer-aided design (CAD), can generate three-dimensional structures layer by layer in a variety of materials. Currently, there are mainly four kinds of 3D printing technologies, Fused Deposition Modeling (FDM), Laminated Object Manufacturing (LOM), Stereo Lithography Apparatus (SLA), Selective Laser Sintering (SLS), applied in the manufacture of bone tissue engineering scaffolds, among which FDM has been widely used in bone tissue engineering due to its application in a variety of thermoplastic polymer materials, convenience in use and low cost [17,18]. Fused Deposition Modeling (FDM) is a common scaffold fabrication technique based on material extrusion: a heated extrusion head extrudes thermoplastic fiber material and deposits a semi-fused polymer layer by layer on a heated platform. Because of the fusion between layers, the scaffolds manufactured by FDM have good structural integrity and mechanical properties. Among many 3D-printed porous calcium phosphate–polymer scaffolders, PCL/β-TCP composite material is the most interesting and studied one [19].

PCL is a thermoplastic polymer with excellent biocompatibility [20,21]. Its low melting point (60 °C) and high decomposition point (350 °C) make it an ideal thermoplastic material for hot extrusion technology [18,22]. The degradation time is less than 2 years [23,24], and the degradation products are non-toxic carbon dioxide and water, which can be absorbed by the human body. β-TCP has good biocompatibility and bone conductivity. Its dissolution will release calcium ions, which contribute to osteogenic differentiation [25], but it has fast degradation in vivo, limited mechanical strength and high brittleness [26]. The combination of the two materials takes advantage of the toughness of PCL and the stiffness of TCP and is more in line with the mechanical property requirements of bone scaffold materials [20,27,28,29,30,31]. The introduction of finite element method (FEM) has greatly improved the methodology in the design process of biomedical applications. The combination of FEM and 3D bioprinting technology not only solves the design and optimization problems of 3D bioprinting scaffolds but also helps to predict the performance of scaffolds under various physiological conditions and analyze these behaviors [32,33,34,35,36]. In recent years, exciting results have been achieved by using FEM to optimize the design and predict the mechanical behavior of stents in specific tissue applications [37,38,39,40,41,42]. Ryan et al. used finite element analysis to evaluate the mechanical properties of sintered titanium powder with different porosity ranges for the first time. The finite element analysis results were compared with the experimental results, and it was found that cell modeling could accurately predict the mechanical properties of scaffolds. The finite element analysis results are slightly lower than the physical prototype. This geometric variation will be reduced if the manufacturing process is improved during the conversion of CAD models to prototypes [37]. Kim et al. [38] used finite element analysis to investigate the use of composite bone plates in the healing of long bone fractures, such as transverse fractures of the tibia, taking into account contact conditions and changes in callus material properties associated with the healing period. ABAQUS 6.71 was used to study changes in stress distribution with changes in axial and compressive loads, as well as changes in material properties over time. Two composite bone plates were considered in the analysis, common braided epoxy/carbon composite and BCP/Kevlar composite, both of which are biocompatible. The relationship between stress distribution and bone plate alignment and healing time was analyzed by finite element method. The results showed that the composite bone plates with stacked sequences in Kevlar/BCP composites produced the most appropriate strain distribution at the fracture site during the early healing process and reduced the stress shielding effect between bone and plate. Badge et al. [39] studied various geometric design parameters of an extruded 3D printed stand. Based on the finite element analysis of 36 scaffolds, the porosity and Young’s modulus of the composites are predicted. The results show that the porosity and mechanical stability of β-TCP scaffolds can be determined by analyzing the width of the strut and the diameter of the nozzle. Based on the finite element analysis results, the best mechanical properties of the support were proposed. Tagliabue et al. [40] obtained 3D models from micro-CT scans and used finite element analysis techniques to find the elastic coefficients of bioactive glass scaffolds. The calculation results of the finite element model show that the calculation results of the finite element model are in agreement with the existing ones, and the relationship between the elastic properties, porosity and wall thickness is established. Askari et al. [41] investigated the mechanical properties of zirconia scaffolds using a finite element model based on micro-CT. The stress distribution, plastic strain and flow stress of the zirconia support were calculated and analyzed. Using the proposed method, the relationship between macroscopic behavior and microscopic characteristics of scaffolds can be deeply understood. Caiazzo et al. [42] compared the simulation results with actual results from quasi-static compression tests and referenced similar studies on other lightweight structures in the literature to measure the model’s effectiveness. The virtual volume is reduced to 1/8, effectively reducing the total time to solve the problem by more than 90%; compared with the similar simulation studies of other lightweight structures in the literature, the mismatch between the actual mechanical properties and the simulated values is low. These biases are due to an incomplete database or local imperfections of the actual sample due to typical LPBF limitations.

All the above studies indicate that finite element method is indeed an effective tool to study the effect of scaffold design on the mechanical properties of bone scaffolds. Tissue-specific scaffolds can be identified through FEM’s capabilities in design, analysis and prediction, an approach that may accelerate the rate of progress in bioengineered tissue construction using 3D bioprinting. Despite this, there is not much research on the verification analysis between simulation and real experiments, and there are still significant gaps between the two, including the influence of manufacturing processes. Therefore, people still need to explore further and find solutions.

In this study, the porcine cancellous bone structure was used as the prototype. The porosity and thickness were extracted as the bionic design parameters, and the porosity and structural shape were used as the difference indexes to design the bionic bone trabecular scaffold. Finite element method (FEM) was used to test the mechanical properties of the designed bone trabecular model. The PCL/β-TCL porous scaffold was prepared by FDM method, and the mechanical properties were tested. The FEM simulation results were compared and verified, and the influences of porosity and pore shape on mechanical properties were analyzed, which provided reference for the mechanical properties of PCL/β-TCP porous biomimetic scaffold materials in the application of bone scaffolders.

## 2. Materials and Methods

### 2.1. Parameter Extraction of Cancellous Bone and Biomimetic Scaffold Model Establishment

#### 2.1.1. Parameter Extraction of Cancellous Bone

**Figure 1:**
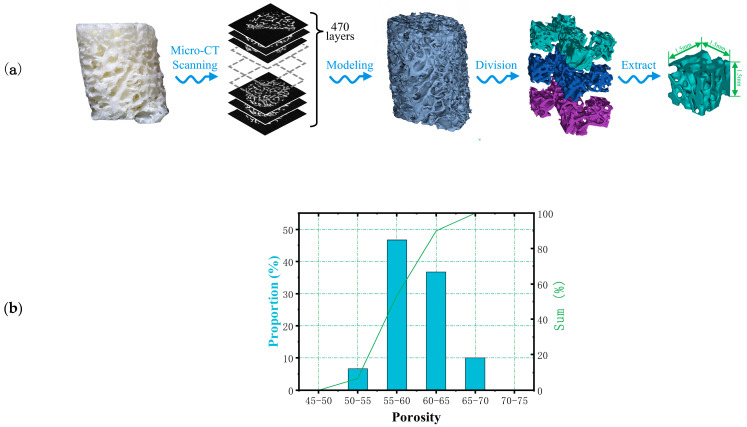
**(a)** Extraction process and (**b**) the proportion of pores in bone trabecular unit model.

In this study, Micro-CT (Inveon Multimodality System, Siemens Medical Solutions, Malvern, PA, USA) was used for microstructural analysis of cancellous bone samples collected from the rib area of pigs, which were obtained from local commercial slaughterhouses. Figure 1 shows the extraction process of related parameters of bone trabeculae. The selected bone trabeculae samples were divided into three layers for extraction and then divide each layer into five 2 mm sided cubes. A separate color was assigned to each segmented model for later statistical analysis. A total of 10 bone trabecular models were selected and 150 samples were extracted for reconstruction and parameter measurement. The porosity of each model was counted in the range of 40% to 80% in 5% increments. The results were shown in Figure 1. The results showed that the average porosity of the block mode l was 58.9%, which was concentrated in the range of 55–65%, accounting for 83.4% of the total samples. Therefore, in this study, the porosity of the bionic scaffold was selected as 55–65%, and the porosity of the test model was determined to be 55%, 57%, 59%, 61%, 63%, 65% at the interval of 2%. The average thickness of bone trabeculae in the region was calculated to be 0.209 ± 0.004 mm.

#### 2.1.2. Bionic Bone Scaffold Model Building

**Figure 2:**
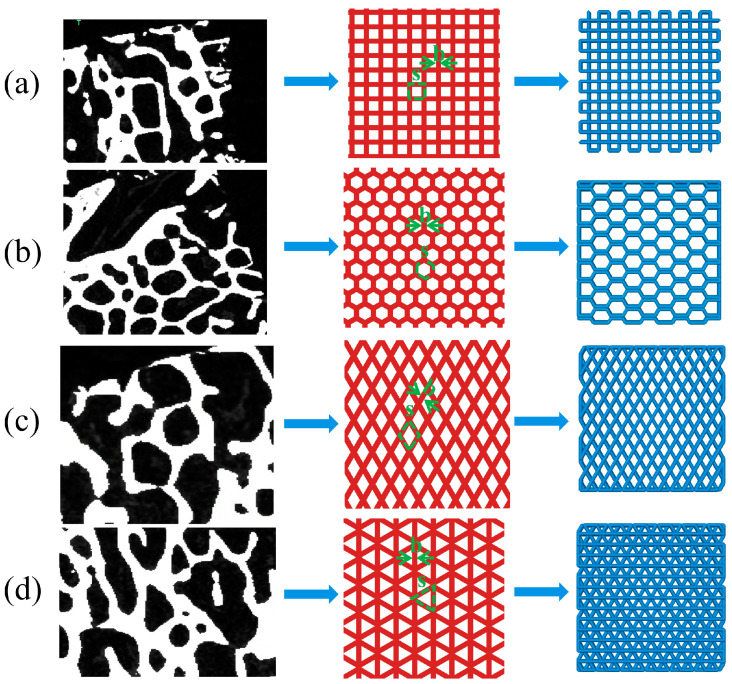
Extraction and arrange ment of bionic bone scaffold filling structure (**a**) rectangle (**b**) honeycomb (**c**) diamond (**d**) triangle.

As shown in Figure 2, four structures, namely, rectangle, triangle (with three equal sides), honeycomb and diamond (with 60° Angle), were extracted from the bone trabecular structure as scaffold-filling structures. Using SolidWorks software, the porosity of bone trabeculae was 55–65% and the average thickness was 0.2 mm as bionic design parameters. The completed model was generated into an STL (Stereolithography) format file, which was used for the following finite element analysis and 3D printing.

### 2.2. Simulation of Mechanical Properties of Bionic Bone Scaffold Model

In order to determine the mechanical behavior of four selected structural bone trabecular scaffolders under static loads, the compression and bending tests were simulated using Abaqus 6.14 (Dassault System, SIMULIA, Paris, France). Select unit size was 6 × 6 × 2 mm trabecular bone scaffolds as the compression test and bending test model. The porosity of the scaffold was 55%, 57%, 59%, 61%, 63% and 65%, respectively. Since there were many holes in the bone trabecular scaffold model, the automatic meshing function of ABAQUS could not guarantee the validity of the partitioning results. Therefore, HyperMesh was used to mesh the designed bone trabecular scaffold model. Hexahedral elements of the same size were used in all the grid components of the scaffold model to eliminate the influence of grid sensitivity on the results. As shown in Table 1, it can be seen that the standard deviation (Std. dev) maximum of the mesh number of compressed samples was 1647.929, and Coefficient of Variation (COV) was 2.252%. The difference of the overall mesh division results was small and had good statistical significance. The processed mesh components were imported into ABAQUS, and the material parameters of bone trabeculae were determined by referring to relevant literature, as shown in Table 2 [43,44].

**Figure 3:**
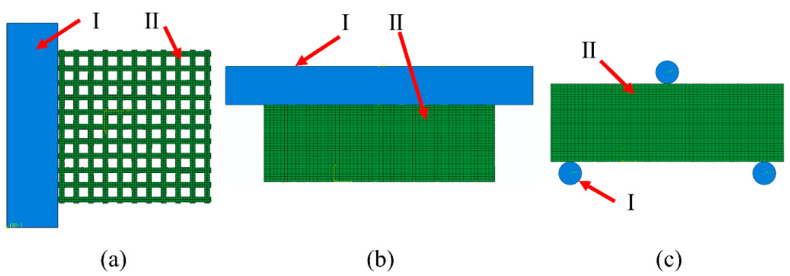
ABAQUS compression experimental assembly drawing where I is the rigid body and II is the samples: (**a**) Transverse compression; (**b**) axial compression; (**c**) three-point bending experimental assembly drawing.

The compression experiment was divided into two parts: transverse compression experiment and axial compression experiment. The assembly relationship was shown in Figure 3a,b. Horizontal compression experiments were conducted using rigid body components for horizontal compression of samples: reference points were set on rigid body compression components, which were combined with rigid body through the coupling method. Contact between compression parts and samples was controlled through surface-to-surface interaction, and the internal self-contact of sample mesh was added through general contact. A displacement of 1.5 mm was applied to the reference point to limit the free degree of the sample’s compression surface. After the simulation, the resistance and displacement curves of reference point RP were output.

The three-point bending test is shown in Figure 3c. It was carried out on samples through cylindrical components. The coupling method was used to combine three reference points RP1–3 with three cylindrical rigid bodies (D = 0.6 mm), which limited the freedom of the cylindrical parts at the bottom. A 1 mm vertical downward displacement was applied to the top cylindrical parts. The interactions of self-contact between grids and face-to-face contact between rigid bodies and mesh components were considered. The distance between the bottom two parts was 5 mm.

### 2.3. Preparation of β-TCP/PCL Scaffold

The β-TCP/PCL powder was mixed by a rotating planetary ball mill (QXQM-8, Changsha Tianchuang Powder Technology Co., Ltd., Changsha, China), The mixed powder consisted of 30 wt.% β-TCP. Subsequently, a composite wire with a diameter of 1.75 mm was prepared by wire extrusion mechanism. The heating temperature was 80 °C. The extrusion speed was 5 mm/s. A 3D printer (DK2, Flash Technology, Shenzhen, China) was used to melt and extruder the processed wire to prepare the sample. The printing speed was 2 mm/s, the printing temperature was 180 °C and cooling was by blowing air during extrusion.

### 2.4. Mechanical Properties Testing of Samples

The elastic modulus and yield strength of the material were obtained by compression and bending tests. The size of compression test sample prepared by 3D printing was 10 × 10 × 10 mm, and the bending test sample was 10 × 25 × 2 mm, with 6 samples in each group. Electronic universal testing machine (C43.104, MTS Systems Co., Ltd., Canton, OH, USA) was used to test the mechanical properties, and the constant displacement rate of the indenter was 1 mm/min.

## 3. Results and Discussion

### 3.1. Simulation Analysis of Structural Mechanical Properties of Bionic Bone Scaffold

#### 3.1.1. Analysis of Transverse Compression Test Simulation Results

**Figure 4:**
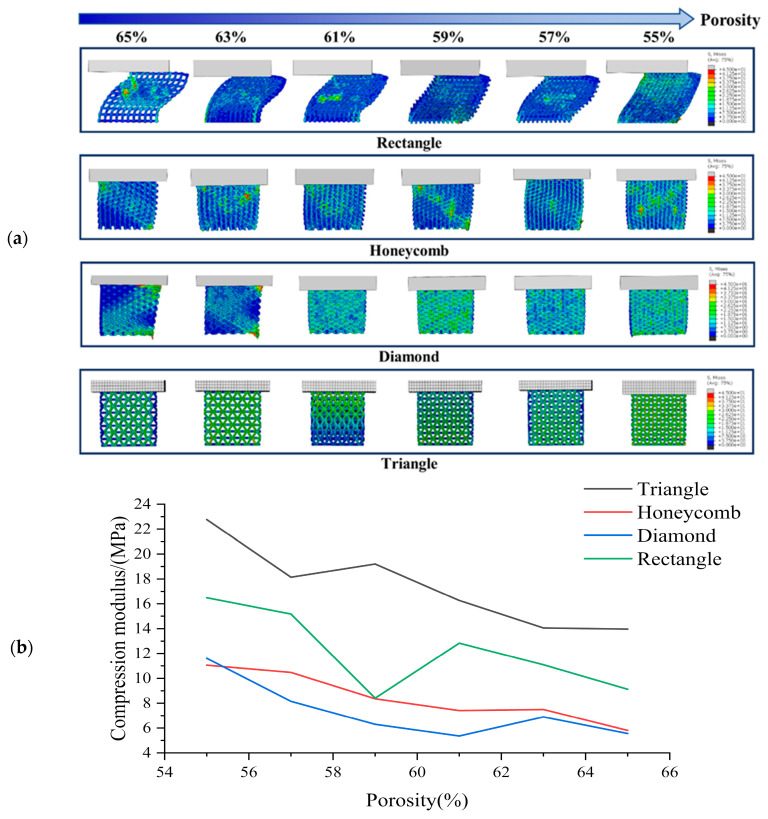
(**a**) Transverse compression test simulation results and (**b**) Compression modulus of each sample in transverse compression test with different porosity.

The simulation results of transverse compression test are shown in Figure 4. As can be seen from Figure 4, the deformation of square holes’ samples was large in the compression process, and there was an obvious lateral buckling phenomenon, and the buckling distance gradually decreases with the porosity’s reduction. By observing the simulation results of honeycomb hole samples, we can see that compared with square hole samples, the honeycomb hole samples have stronger stability. The honeycomb hole samples had less lateral buckling phenomenon, and the stress distribution was wider. The results showed that the honeycomb specimens could conduct stress more effectively. According to the simulation results of rhomboid hole samples, the compressive deformation of the rhomboid hole was the largest because of its high deformability. In the compression results, the phenomenon that the pores’ upper wall has contacted with the bottom one means that the support model cannot play a supporting role. Generally, the sample with rhomboid holes did not have the ability to resist the compression load. It can be seen from the simulation results of the triangular hole sample that it has strong stability [45]. The overall compression displacement was minimal, and the stress distribution was more uniform and wider. There was no obvious stress concentration in all the samples.

Based on Figure 4, it is evident that the compressive strength of the scaffold decreases gradually with increasing porosity 46]. The compressive strength rankings for all sample shapes remain consistent across each porosity level, with the Triangle exhibiting the highest compressive strength under lateral compression, followed by the Rectangle, Honeycomb and Diamond. These findings suggest that the Triangle shape has the best lateral compressive performance. While the compressive strength of the Rectangle shape is greater than that of the Honeycomb, its stability is typically lower due to greater lateral buckling during compression. Our analysis indicates that the triangle shape has a lateral compressive modulus 1.06 times higher than that of the honeycomb, 1.38 times higher than that of the diamond and 0.42 times higher than that of the square.

#### 3.1.2. Analysis of Axial Compression Test Simulation Results

**Figure 5:**
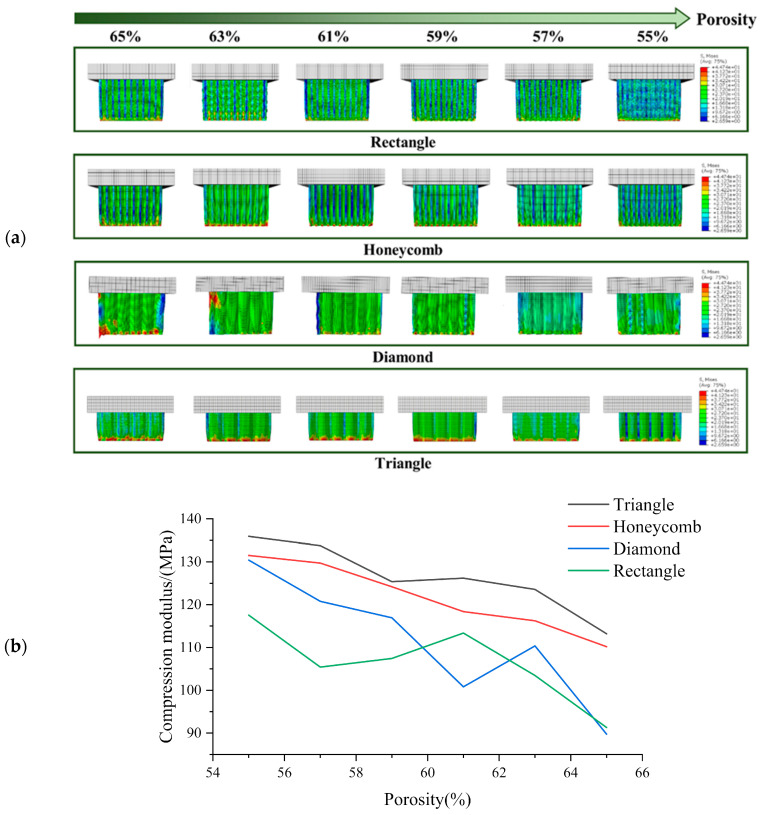
(**a**) Axial compression test simulation results and (**b**) Compression modulus of each sample in axial compression test with different porosity.

The simulation results of axial compression test are shown in Figure 5. According to the axial compression simulation results of the square hole sample, when the porosity was high, the square sample appeared as a relatively obvious wavy, and at this time, the bionic scaffold lost the holding ability. When the porosity was low, the bending phenomenon of the sample was small, and the overall stress was staggered. According to the axial compression simulation results of honeycomb hole samples, there is no obvious bending phenomenon in the case of low or high porosity. This indicates that honeycomb hole samples have good structural stability. The overall axial compression displacement of the sample was small. By observing the axial compression simulation results of the samples with triangular holes, it can be seen that these samples have strong stability. Under different porosity conditions, the compression deformation and displacement of the samples are small, and the pore shape has no obvious deformation. The stress distribution was uniform. It shows that the triangular hole sample has excellent resistance to axial compression load [45]. According to the axial compression simulation results of the rhomboid-hole sample, the bearing part of the sample would distort and deform under the condition of high porosity. This indicates that the structural stability of the sample has been lost. The phenomenon of stress concentration is obvious, which leads to the rhomboid hole sample’s easy to collapse failure during actual compression. It shows that the resistance of rhomboid hole samples to axial compression is poor.

The compressive strength of all specimens gradually decreases with the increase of porosity [46], as illustrated in Figure 5. Among all porosities, the axial compressive strength is found to be the highest in the Triangle samples, followed by the Honeycomb. The compressive strength of the Diamond and Rectangle varies with different porosities, in agreement with the phenomenon depicted in Figure 5. These findings indicate that the Triangle shape exhibits an axial compressive strength that is 0.038 times higher than that of the Honeycomb, 0.133 times higher than that of the Diamond and 0.187 times higher than that of the Rectangle.

#### 3.1.3. Analysis of Three-Point Bending Simulation Test Results

**Figure 6:**
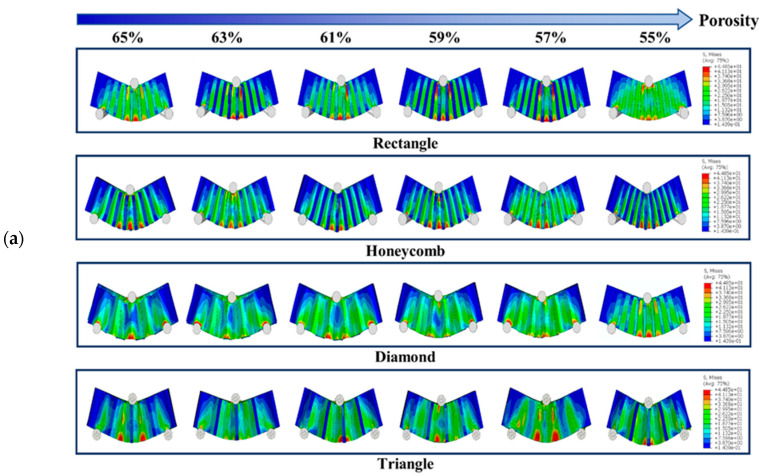

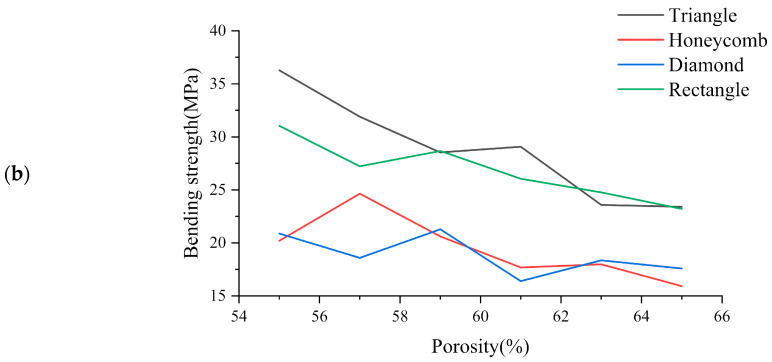
(**a**) Three-point bending test simulation results and (**b**) Three-point bending results of each sample with different porosity.

Figure 6 shows the simulation test results of three-point bending for different samples with different porosity. It can be seen from the observation that when the porosity of the square hole samples decreased gradually, their deformation degree decreased gradually. However, compression phenomenon appeared locally lead to the failure of the top unit. The number of high-stress units generated in the bearing part was small, indicating that this part was too short to carry on this work. When the honeycomb samples were pressed, the samples themselves were not being pressed in, which indicates that they have strong stress transfer ability. The maximum stress range of the bearing part was larger than the square hole samples, so that the load bearing ability would be enhanced correspondingly. The simulation results of triangular hole samples show that the high stress was mainly concentrated in the tensile region of the bearing end, and the overall bending strength was high. It is thought that due to their stable structural characteristics, the simulation results of the samples with diamond holes show that the overall stress distribution of the samples were more uniform. There was no obvious phenomenon of stress concentration in the process of cylinder pressing because of the high deformability of diamond holes. However, the deformation degree was relatively large. Under the action of pressing load, the bending of the samples was obvious, which means that the resistance to deformation was poor.

The bending strength diagram for the bracket is presented in Figure 6. It can be observed that the bending strength gradually decreases as the porosity increases [3]. Specifically, the Triangle and Rectangle samples exhibit higher bending strength, with the former performing significantly better than the latter at low porosity. However, as the porosity increases to 62%, the bending strength of the Rectangle samples becomes superior to that of the Triangle samples. On the other hand, the Honeycomb and Diamond samples display poor bending strength. The results indicate that the bending strength of Triangle samples increases 0.476 times, 0.528 times and 0.07 times compared to that of Honeycomb, Diamond and Rectangle samples, respectively.

### 3.2. Performance Characteristics of 3D Printed β-TCP/PCL Scaffold

The viscosity, solid load and stability of FDM printed wire are important for the preparation of high performance porous scaffold. In this study, the slurry viscosity increased with the increase of β-TCP content in PCL matrix. When the content of β-TCP in PCL was greater than 30wt%, the raw material powder was difficult to disperse evenly. The slurry fluidity was poor, easy to block the nozzle, and it was difficult to meet the requirements of extrusion molding. When the content of β-TCP was less than 30%, the extrusion line was stable and continuous, and the molding was uniform, suitable for printing. The higher the TCP content, the better the biological activity of the material [47,48]. Therefore, the maximum filling volume of β-TCP was selected as 30%wt in this study, similar to the results of previous studies [49].

**Figure 7:**
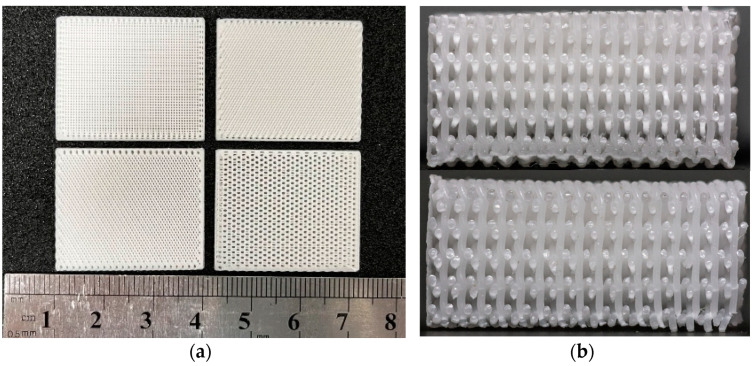
Photo of 3D printed bionic scaffold samples: (**a**) Photo of the pore shape of the samples; (**b**) Profile view of the print samples.

Figure 7a shows the biomimetic bracket with 30%β-TCP content prepared. The printed parts have uniform texture and the side appearance c arranged in bundles. In Figure 7b, it can be seen that the internal plane of the print is flat and the wire section is evenly distributed. This indicates that the overall scaffolds pore size and morphology are uniform and can maintain a certain connectivity. The printed porous β-TCP/PCL scaffolds have good formability, which indicates that it is feasible to print the scaffolds using FDM 3D printing technology [50,51].

**Figure 8:**
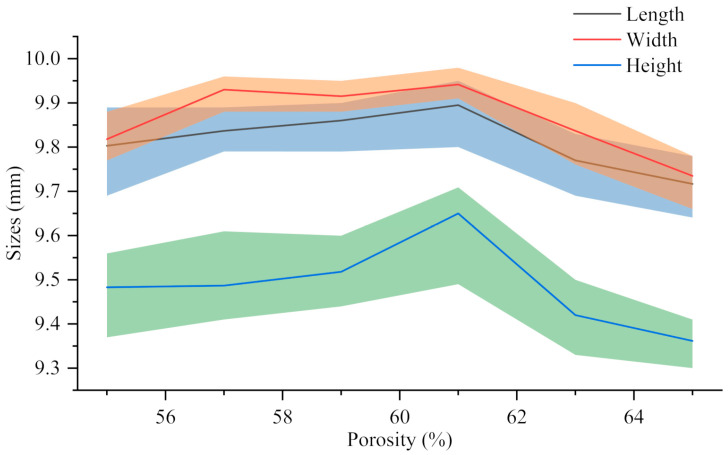
The actual size of 30%β-TCP/PCL scaffolds with different porosity.

Shrinkage rate *η* refers to the percentage between the design size of the sample *V_v_* and the actual size *V_r_* cooled to room temperature after printing, which reflects the size reduction degree of the sample after processing and cooling. The calculation formula is shown in Equation (1). The shrinkage rate of each support in Figure 8 in different directions (9.77 ± 0.09 mm long, 9.84 ± 0.09 mm wide, 9.39 ± 0.13 mm high) is calculated. The results show that the length is 2.33 ± 0.92%, the width is 1.61 ± 0.88% and the height is 6.12 ± 1.30%. Compared with the length and width, the shrinkage rate of the height is more obvious, which may be related to the effect of gravity in the printing and cooling process of the material. However, they are all within 8%, which ensures the integrity of the scaffold. It has proved that FEM technology can be used in the fabrication process of bionic scaffold.

(1) $$\eta =\left(V_{v}-V_{r}\right)/V_{v}\times 100\%$$

### 3.3. Mechanical Properties of 3D Printed β-TCP/PCL Scaffold

**Figure 9:**
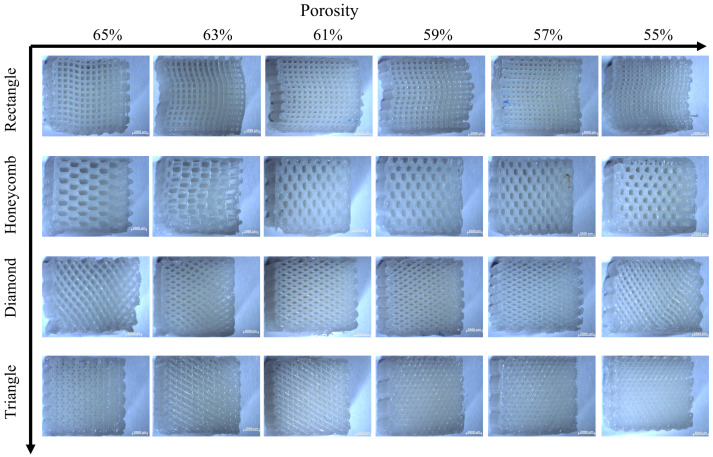
Transverse compression deformation maps of four samples with different porosity.

**Figure 10:**
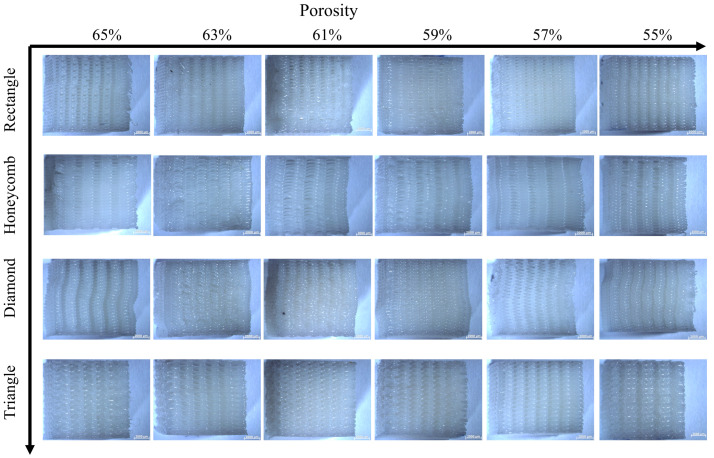
Deformation figure in axial compression direction of four samples with different porosity.

The stereomicroscope (ZEISS Stereo Discovery. V20, Carl Zeiss, Oberkochen, Germany) was used to photograph the deformation of the compressed sample after the test. Figure 9 and Figure 10 show the results of lateral and axial compression of the sample block. As can be seen from Figure 9, the square sample has distortion in the compression process, and the degree of deformation decreases with the decrease of porosity. Most of the distortion parts occur in the middle of the sample, indicating that its support is too fragile to bear a large lateral load. There is no obvious distortion and deformation of honeycomb samples after transverse compression test, and the honeycomb structure can ensure the stability of the overall structure in the compression process. But the honeycomb pore has changed from regular hexagon to nearly rectangular, which will lead to the loss of structural stability. It can be seen from the observation of the rhomboid structure samples that a large “collapse” phenomenon occurs inside the structure when the porosity is 65%, and a slight distortion occurs when the porosity is low. The triangular sample is generally stable, and it still maintains considerable stability with high porosity, while the pore shape does not change greatly. The overall test results fit well with the simulation results, which proves the effectiveness of FEM setting.

As for the axial compression test, the following conclusions can be drawn from the observation in Figure 10: compared with the transverse compression, the axial compression samples have a smaller deformation degree. Square and triangular void samples have no obvious deformation, and they maintain a better supporting capacity in the compression process. The axial compression samples with honeycomb pores show torsional bending phenomenon at the boundary at high porosity, which indicates that the bearing capacity of the samples is damaged greatly, and there is no obvious deformation phenomenon at low porosity, which means great stability. The overall stability of rhomboid samples is poor, and the obvious torsion phenomenon occurs at both low and high porosity, indicating that the stability is poor.

**Figure 11:**
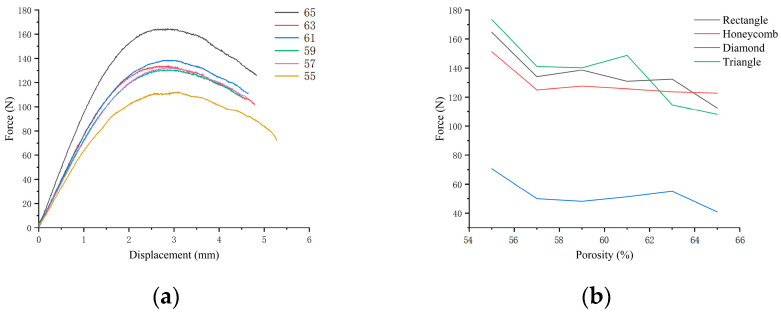
(**a**) Three-point bending resistance–displacement diagram of square pore with different porosity; (**b**) Ultimate bearing resistance diagram in three-point bending test of four samples with different porosity.

For the three-point bending sample, the diagram of resistance and compression displacement in the bending process is shown in Figure 11. The resistance of the sample in the pressing process at the upper contact point increases rapidly with the compression displacement. When the displacement exceeds this limit, the supporting force gradually decreases. The ultimate bearing forces of the four pore structure samples under different porosity conditions can be obtained in Figure 11. The ultimate bearing capacity of triangular samples under 59%, 61%, 63% and 65% porosity conditions were the largest among the four samples, while the ultimate bearing capacity under 55% and 57% porosity conditions is smaller than honeycomb and square. The bearing capacity of the pores of the rhomboid structure is the worst, and there is an obvious gap between them and the other three structures.

## 4. Conclusions

In this study, the porous tricalcium phosphate (TCP)/polycaprolactone (PCL) composite scaffold was successfully prepared by FDM 3D printing technology for cancellous bone tissue engineering. The optimum content of TCP is less than 30wt%. The simulation results of transverse compression and axial compression were verified by experiments, and the validity of the simulation results was proved. The results showed that FEM was effective in predicting structural stability. The simulation and experiment showed that the triangular pore structure had strong stability in compression and three-point bending process. However, it is important to acknowledge that the study only focused on four specific pore structures and further research is necessary to investigate the stability of different pore geometries and sizes. Further research is necessary to validate and optimize the results and to explore the potential of other scaffold designs and materials.

## Figures and Tables

**Table 1:** The number of grids of each simulation component.

Number	Grid Number	Number	Grid Number	Number	Grid Number	Number	Grid Number	Std. Dev	COV (%)
Fang-55	71,150	Ling-55	75,570	San-55	72,280	Feng-55	73,650	1647.929	2.252
Fang-57	71,080	Ling-57	73,910	San-57	71,820	Feng-57	73,540	1174.508	1.618
Fang-59	70,560	Ling-59	72,830	San-59	71,860	Feng-59	72,400	853.1522	1.186
Fang-61	70,560	Ling-61	69,640	San-61	68,700	Feng-61	68,360	858.1812	1.238
Fang-63	67,630	Ling-63	68,010	San-63	68,270	Feng-63	67,790	240.5722	0.354
Fang-65	68,860	Ling-65	67,110	San-65	67,690	Feng-65	67,400	664.624	0.981

**Table 2:** Material properties of bone trabeculae.

Density (Toone/mm^3^)	Modulus of Elasticity (MPa)	Failure Stress (MPa)	Failure Strain	Poisson’s Ratio
3.15 × 10^−9^	223	45	2.23	0.3

## Data Availability

The data presented in this study are available on request from the corresponding author.

## References

[1] Glowacki J. (1988). The Law of Bone Remodelling. Plast. Reconstr. Surg..

[2] Finkemeier C.G. (2002). Bone-Grafting and Bone-Graft Substitutes. J. Bone Jt. Surg. Am..

[3] Mishra R., Bishop T., Valerio I.L., Fisher J.P., Dean D. (2016). The potential impact of bone tissue engineering in the clinic. Regen. Med..

[4] Winkler T., Sass F.A., Duda G.N., Schmidt-Bleek K. (2018). A review of biomaterials in bone defect healing, remaining shortcomings and future opportunities for bone tissue engineering: The unsolved challenge. Bone Jt. Res..

[5] Amini A.R., Laurencin C.T., Nukavarapu S.P. (2012). Bone Tissue Engineering: Recent Advances and Challenges. Crit. Rev. Biomed. Eng..

[6] Muschler G.F., Nakamoto C., Griffith L.G. (2004). Engineering principles of clinical cell-based tissue engineering. J. Bone Jt. Surg..

[7] Rainer A., Giannitelli S.M., Accoto D., De Porcellinis S., Guglielmelli E., Trombetta M. (2012). Load-Adaptive Scaffold Architecturing: A Bioinspired Approach to the Design of Porous Additively Manufactured Scaffolds with Optimized Mechanical Properties. Ann. Biomed. Eng..

[8] Rose F.R., Cyster L.A., Grant D.M., Scotchford C.A., Howdle S.M., Shakesheff K.M. (2004). In vitro assessment of cell penetration into porous hydroxyapatite scaffolds with a central aligned channel. Biomaterials.

[9] Damadzadeh B., Jabari H., Skrifvars M., Airola K., Moritz N., Vallittu P.K. (2010). Effect of ceramic filler content on the mechanical and thermal behaviour of poly-l-lactic acid and poly-l-lactic-co-glycolic acid composites for medical applications. J. Mater. Sci. Mater. Med..

[10] Senatov F.S., Niaza N.K., Zadorozhnyy M.Y., Maksimkin A.V., Kaloshkin S.D., Estrin Y.Z. (2016). Mechanical properties and shape memory effect of 3D-printed PLA-based porous scaffolds. J. Mech. Behav. Biomed. Mater..

[11] Simpson R., Nazhat S., Blaker J., Bismarck A., Hill R., Boccaccini A., Hansen U., Amis A. (2015). A comparative study of the effects of different bioactive fillers in PLGA matrix composites and their suitability as bone substitute materials: A thermo-mechanical and in vitro investigation. J. Mech. Behav. Biomed. Mater..

[12] Xu N., Ye X., Wei D., Zhong J., Chen Y., Xu G., He D. (2014). 3D Artificial Bones for Bone Repair Prepared by Computed Tomography-Guided Fused Deposition Modeling for Bone Repair. ACS Appl. Mater. Interfaces.

[13] Thavornyutikarn B., Chantarapanich N., Sitthiseripratip K., Thouas G.A., Chen Q. (2014). Bone Tissue Engineering Scaffolding: Computer-Aided Scaffolding Techniques. Prog. Biomater..

[14] Bajaj P., Schweller R.M., Khademhosseini A., West J.L., Bashir R. (2014). 3D Biofabrication Strategies for Tissue Engineering and Regenerative Medicine. Annu. Rev. Biomed. Eng..

[15] Murphy S.V., Atala A. (2014). 3D bioprinting of tissues and organs. Nat. Biotechnol..

[16] Zadpoor A.A., Malda J. (2017). Additive Manufacturing of Biomaterials, Tissues, and Organs. Ann. Biomed. Eng..

[17] Serra T., Ortiz-Hernandez M., Engel E., Planell J.A., Navarro M. (2014). Relevance of PEG in PLA-Based Blends for Tissue Engineering 3D-Printed Scaffolds. Mater. Sci. Eng. C.

[18] Zein I., Hutmacher D.W., Tan K.C., Teoh S.H. (2002). Fused deposition modeling of novel scaffold architectures for tissue engineering applications. Biomaterials.

[19] Babilotte J., Guduric V., Le Nihouannen D., Naveau A., Fricain J.-C., Catros S. (2019). 3D printed polymer–mineral composite biomaterials for bone tissue engineering: Fabrication and characterization. J. Biomed. Mater. Res. Part B Appl. Biomater..

[20] Hutmacher D.W., Schantz T., Zein I., Ng K.W., Teoh S.H., Tan K.C. (2001). Mechanical properties and cell cultural response of polycaprolactone scaffolds designed and fabricated via fused deposition modeling. J. Biomed. Mater. Res..

[21] Corden T., Jones I., Rudd C., Christian P., Downes S., McDougall K. (2000). Physical and biocompatibility properties of poly-ε-caprolactone produced using in situ polymerisation: A novel manufacturing technique for long-fibre composite materials. Biomaterials.

[22] Olubamiji A.D., Izadifar Z., Si J.L., Cooper D.M.L., Eames B.F., Chen D.X. (2016). Modulating mechanical behaviour of 3D-printed cartilage-mimetic PCL scaffolds: Influence of molecular weight and pore geometry. Biofabrication.

[23] Kim B.S., Jang J., Chae S., Gao G., Kong J.-S., Ahn M., Cho D.-W. (2016). Three-dimensional bioprinting of cell-laden constructs with polycaprolactone protective layers for using various thermoplastic polymers. Biofabrication.

[24] Kim J., McBride S., Tellis B., Alvarez-Urena P., Song Y.-H., Dean D.D., Sylvia V.L., Elgendy H., Ong J., Hollinger J.O. (2012). Rapid-prototyped PLGA/β-TCP/hydroxyapatite nanocomposite scaffolds in a rabbit femoral defect model. Biofabrication.

[25] Rai B., Lin J.L., Lim Z.X., Guldberg R.E., Hutmacher D.W., Cool S.M. (2010). Differences between in vitro viability and differentiation and in vivo bone-forming efficacy of human mesenchymal stem cells cultured on PCL–TCP scaffolds. Biomaterials.

[26] Santos C.F., Silva A.P., Lopes L., Pires I., Correia I.J. (2012). Design and production of sintered β-tricalcium phosphate 3D scaffolds for bone tissue regeneration. Mater. Sci. Eng. C.

[27] Kretlow J.D., Mikos A.G. (2007). Review: Mineralization of Synthetic Polymer Scaffolds for Bone Tissue Engineering. Tissue Eng..

[28] Schantz J.-T., Hutmacher D.W., Lam C.X.F., Brinkmann M., Wong K.M., Lim T.C., Chou N., Guldberg R.E., Teoh S.H. (2003). Repair of Calvarial Defects with Customised Tissue-Engineered Bone Grafts II. Evaluation of Cellular Efficiency and Efficacy in vivo. Tissue Eng..

[29] Shor L., Güçeri S., Wen X., Gandhi M., Sun W. (2007). Fabrication of three-dimensional polycaprolactone/hydroxyapatite tissue scaffolds and osteoblast-scaffold interactions in vitro. Biomaterials.

[30] Wang F., Shor L., Darling A., Khalil S., Sun W., Güçeri S., Lau A. (2004). Precision extruding deposition and characterization of cellular poly-ε-caprolactone tissue scaffolds. Rapid Prototyp. J..

[31] Woodruff M.A., Hutmacher D.W. (2010). The return of a forgotten polymer—Polycaprolactone in the 21st century. Prog. Polym. Sci..

[32] Baldino L., Naddeo F., Cardea S., Naddeo A., Reverchon E. (2015). FEM modeling of the reinforcement mechanism of Hydroxyapatite in PLLA scaffolds produced by supercritical drying, for Tissue Engineering applications. J. Mech. Behav. Biomed. Mater..

[33] Ostrowska B., Di Luca A., Moroni L., Swieszkowski W. (2016). Influence of internal pore architecture on biological and mechanical properties of three-dimensional fiber deposited scaffolds for bone regeneration. J. Biomed. Mater. Res. Part A.

[34] Boccaccio A., Ballini A., Pappalettere C., Tullo D., Cantore S., Desiate A. (2011). Finite Element Method (FEM), Mechanobiology and Biomimetic Scaffolds in Bone Tissue Engineering. Int. J. Biol. Sci..

[35] Eshraghi S., Das S. (2012). Micromechanical finite-element modeling and experimental characterization of the compressive mechanical properties of polycaprolactone–hydroxyapatite composite scaffolds prepared by selective laser sintering for bone tissue engineering. Acta Biomater..

[36] Barui S., Chatterjee S., Mandal S., Kumar A., Basu B. (2017). Microstructure and compression properties of 3D powder printed Ti-6Al-4V scaffolds with designed porosity: Experimental and computational analysis. Mater. Sci. Eng. C.

[37] Ryan G., McGarry P., Pandit A., Apatsidis D. (2009). Analysis of the mechanical behavior of a titanium scaffold with a repeating unit-cell substructure. J. Biomed. Mater. Res. Part B Appl. Biomater..

[38] Kim S.-H., Chang S.-H., Jung H.-J. (2010). The finite element analysis of a fractured tibia applied by composite bone plates considering contact conditions and time-varying properties of curing tissues. Compos. Struct..

[39] Bagde A.D., Kuthe A.M., Nagdeve S.R., Dahake S.W., Sapkal P.S., Daronde S.B., Lande N.H., Sarode B.D. (2019). Geometric Modeling and Finite Element Simulation for Architecture Design of 3D Printed Bio-ceramic Scaffold Used in Bone Tissue Engineering. J. Indian Inst. Sci..

[40] Tagliabue S., Rossi E., Baino F., Vitale-Brovarone C., Gastaldi D., Vena P. (2017). Micro-CT based finite element models for elastic properties of glass–ceramic scaffolds. J. Mech. Behav. Biomed. Mater..

[41] Askari E., Cengiz I., Alves J., Henriques B., Flores P., Fredel M., Reis R., Oliveira J., Silva F., Mesquita-Guimarães J. (2020). Micro-CT based finite element modelling and experimental characterization of the compressive mechanical properties of 3-D zirconia scaffolds for bone tissue engineering. J. Mech. Behav. Biomed. Mater..

[42] Caiazzo F., Guillen D.G., Alfieri V. (2021). Simulation of the Mechanical Behaviour of Metal Gyroids for Bone Tissue Application. Materials.

[43] Bevill G., Eswaran S.K., Gupta A., Papadopoulos P., Keaveny T.M. (2006). Influence of bone volume fraction and architecture on computed large-deformation failure mechanisms in human trabecular bone. Bone.

[44] Langton C.M., Wille M.-L. (2015). Application of ultrasound transit time spectroscopy to human cancellous bone for derivation of bone volume fraction in-vitro. J. Acoust. Soc. Am..

[45] Janmohammadi M., Nourbakhsh M.S., Bahraminasab M., Tayebi L. (2023). Effect of Pore Characteristics and Alkali Treatment on the Physicochemical and Biological Properties of a 3D-Printed Polycaprolactone Bone Scaffold. ACS Omega.

[46] Soufivand A.A., Abolfathi N., Hashemi S.A., Lee S.J. (2020). Prediction of mechanical behavior of 3D bioprinted tissue-engineered scaffolds using finite element method (FEM) analysis. Addit. Manuf..

[47] Dienel K.E.G., van Bochove B., Seppälä J.V. (2019). Additive Manufacturing of Bioactive Poly(trimethylene carbonate)/β-Tricalcium Phosphate Composites for Bone Regeneration. Biomacromolecules.

[48] Blanquer S.B., Gebraad A.W., Miettinen S., Poot A.A., Grijpma D.W., Haimi S.P. (2017). Differentiation of adipose stem cells seeded towards annulus fibrosus cells on a designed poly(trimethylene carbonate) scaffold prepared by stereolithography. J. Tissue Eng. Regen. Med..

[49] Nyberg E., Rindone A., Dorafshar A., Grayson W.L. (2017). Comparison of 3D-Printed Poly-epsilon-Caprolactone Scaffolds Functionalized with Tricalcium Phosphate, Hydroxyapatite, Bio-Oss, or Decellularized Bone Matrix. Tissue Eng. Part A.

[50] Dong Q., Zhang M., Zhou X., Shao Y., Li J., Wang L., Chu C., Xue F., Yao Q., Bai J. (2021). 3D-printed Mg-incorporated PCL-based scaffolds: A promising approach for bone healing. Mater. Sci. Eng. C.

[51] Wang C., Huang W., Zhou Y., He L., He Z., Chen Z., He X., Tian S., Liao J., Lu B. (2020). 3D printing of bone tissue engineering scaffolds. Bioact. Mater..

